# Rate-Dependent Stability and Electrochemical Behavior of Na_3_NiZr(PO_4_)_3_ in Sodium-Ion Batteries

**DOI:** 10.3390/nano14141204

**Published:** 2024-07-16

**Authors:** Marwa Tayoury, Abdelwahed Chari, Mohamed Aqil, Adil Sghiouri Idrissi, Ayoub El Bendali, Jones Alami, Youssef Tamraoui, Mouad Dahbi

**Affiliations:** Materials Science, Energy, and Nano-engineering Department, Mohammed VI Polytechnic University, Ben Guerir 43150, Morocco; marwa.tayoury@um6p.ma (M.T.); abdelwahed.chari@um6p.ma (A.C.); mohamed.aqil@um6p.ma (M.A.); adil.sghiouri@um6p.ma (A.S.I.); ayoub.elbendali@um6p.ma (A.E.B.); jones.alami@um6p.ma (J.A.); youssef.tamraoui@um6p.ma (Y.T.)

**Keywords:** energy storage, NASICON, anode material, sodium-ion batteries (SIBs)

## Abstract

In advancing sodium-ion battery technology, we introduce a novel application of Na_3_NiZr(PO_4_)_3_ with a NASICON structure as an anode material. This research unveils, for the first time, its exceptional ability to maintain high specific capacity and unprecedented cycle stability under extreme current densities up to 1000 mA·g^−1^, within a low voltage window of 0.01–2.5 V. The core of our findings lies in the material’s remarkable capacity retention and stability, which is a leap forward in addressing long-standing challenges in energy storage. Through cutting-edge in situ/operando X-ray diffraction analysis, we provide a perspective on the structural evolution of Na_3_NiZr(PO_4_)_3_ during operation, offering deep insights into the mechanisms that underpin its superior performance.

## 1. Introduction

Energy storage systems are in extremely high demand due to the rapid development of renewable energy sources and electric vehicles. Numerous electrochemical conversion and storage devices have been created in this field [1,2,3]. So far, lithium-ion batteries have received all the attention in the energy storage world. The increasing demand for lithium, coupled with its restricted supply and uneven distribution worldwide, has prompted researchers to seek out alternative elements that can match the performance of lithium metal [4]. Sodium emerges as a viable candidate due to its ample availability throughout the world’s crust, making it one of the most abundant elements on Earth. Moreover, ocean reserves offer an inexhaustible source of sodium [5]. Additionally, being the second lightest and smallest alkali metal after lithium grants sodium batteries significant potential as a groundbreaking solution for energy storage purposes [6,7]. 

Given the wide array of phases suitable for cathode materials in sodium-ion batteries (SIBs), negative electrodes are not commonly found. Presently, hard carbon stands as the predominant choice for SIB anode material, playing a crucial role in demonstrating the feasibility of Na-ion technology [8,9]. However, the potential versus composition profile exhibits a plateau very close to 0 V versus Na^+^/Na, prompting safety concerns at elevated rates due to the risk of sodium plating. Additionally, the low density of carbon (approximately 1.70 g·cm^−3^) results in both a limited volumetric specific capacity and a diminished initial coulombic efficiency (ICE) [10].

Therefore, there is a pressing need for the development of novel anode materials with improved electrochemical performance that can meet the requirements for being used in practical applications. A lot of interest has been devoted to sodium-super-ion conductors (NASICONs) because of their excellent performances, strong redox characteristics, structural stability, and affordable price [11,12,13]. However, the main limitation of these materials is their intrinsic poor electronic conductivity, which significantly impacts their efficiency and rate performance in battery applications. To overcome this challenge, enhancing extrinsic conductivity has become a focal point of research, with carbon structures playing a pivotal role. Various research efforts have demonstrated significant improvements in electronic conductivity and the mitigation of capacity decay and structural collapse employing carbon coating techniques. These enhancements are attributed to the conductive properties of carbon-based materials, such as reduced graphene oxide (rGO) and carbon nanotubes (CNTs), which not only increase the overall conductivity but also prevent particle agglomeration. Furthermore, optimizing the morphology of the carbon used can also improve electrical conductivity. A more ordered structure, as found in graphitic carbon, can facilitate better electron transport compared to amorphous carbon. Adjusting the processing conditions to promote the formation of such structures could be a viable strategy to further enhance the performance of these materials. Additionally, co-doping with elements like nitrogen (N) and Boron (B) has proven successful in creating surface defects that enhance sodium insertion and overall electrochemical performance [14].

Na_3_V_2_(PO_4_)_3_, NaTi_2_(PO_4_)_3_, and NaZr_2_(PO_4_)_3_ have emerged as highly promising contenders among the limited range of NASICON-type materials explored for their potential application as anodes in sodium-ion batteries (SIBs). Their remarkable ability to operate at high rates sets them apart from other candidates. In early reports, Manickam and Takata et al. [15] examined the impact of discharge and charge currents on the initial discharge behavior of cathodes, particularly focusing on enhancing the discharge/charge behavior. Special attention was given to the effect of charge current on the cycle life of rechargeable batteries operating across a broad voltage range of 3.2 to 0.5 V versus Li. The research revealed that higher discharge currents increase the total critical surface area, reducing the concentration of lithium insertion before the cell’s performance becomes limited by diffusion. To mitigate the issues associated with very low discharge rates, such as electrolyte decomposition and leading to low impedance failure, the study concentrated on electrodes with a discharge current of 0.2 mA, suggesting that lowering the charge current could prevent such failures. Importantly, the study found that capacity loss at high discharge rates (0.3 and 0.4 mA) was reversible when switching back to lower discharge rates (0.1 and 0.2 mA), indicating a kinetic origin for the loss of capacity. This is attributed to the movement of a two-phase interface from the particle’s surface to its center, which is a process that can be reversed by decreasing the discharge current. This reversibility suggests that the capacity loss is not due to irreversible electric contact breakage between particles caused by volume changes, offering a promising avenue for optimizing battery performance.

Wang et al. [16] initially reported the use of Na_3_V_2_(PO_4_)_3_ as an anode in sodium half-cells, showing that it achieves a reversible capacity of 170 mAh·g^−1^ across a voltage range of 3.0 to 0.01 V, along with outstanding capacity retention. Furthermore, Jian et al. [17] have shown that Na_3_V_2_(PO_4_)_3_ serves effectively as an anode material for sodium-ion batteries (SIBs), undergoing deep sodiation processes. This involves the formation of Na_4_V_2_(PO_4_)_3_ and Na_5_V_2_(PO_4_)_3_ at voltages of 1.6 V and 0.3 V, respectively, resulting in a significant reversible capacity of 149 mAh·g^−1^ at a current density of 11.7 mA·g^−1^ (0.1 C) and exhibiting strong rate capacities. 

NaTi_2_(PO_4_)_3_ is considered a favorable anode for SIBs as well because of its suitable redox potential (2.1 V vs. Na^+^/Na) and its substantial capacity to accommodate sodium ions, making it a promising choice. The mesoporous NaTi_2_(PO_4_)_3_ nanohybrid-coated substance exhibited a discharge capacity of 102 mAh·g^−1^ when operating at a rate of 0.2 C within the voltage window of 1.0 to 3.0 V vs. Na^+^/Na, as reported in reference [18]. Additionally, when the same material was incorporated into N-doped graphene sheets, it demonstrated an enhanced specific capacity of 129 mAh·g^−1^ at a slower discharge rate of 0.1 C, within the voltage range of 1.5 to 3.0 V vs. Na^+^/Na [19]. The anode material Mg_0.5_Ti(PO_4_)_3_ demonstrated superior rate capability and impressive cycle stability [20]. In the voltage range of 0.01–3.0 V vs. Na^+^/Na, it displays a specific capacity of 268.6 mAh·g^−1^. Recently, an effective carbon coating by CNTs as a conductive network has effectively improved the electrochemical performance and the rate of electron transfer of the Mg_0.5_Ti(PO_4_)_3_ material [20]. On the other hand, Z. Wei [21] explored Ca_0.5_Ti(PO_4_)_3_@C, a nanocomposite that exhibited a high reversible capacity of 264 mAh·g^−1^ and good capacity retention of 75.7% after 1000 cycles at a high current density of 2 A·g^−1^. The electrode exhibits high-rate charge–discharge capabilities due to the underpinning pseudocapacitive effect. Na_3_MnTi(PO_4_)_3_, with its lower redox couple of Ti^2+/3+^ at 0.4 V due to further reduction of titanium, is characterized as an anode for Na-ion batteries. The NMTP/C composite showcases a reversible specific capacity of 200 mAh·g^−1^ at 0.1 C, in a low potential range of 0.01–3.0 V, outperforming the Na_3_V_2_(PO_4_)_3_/C anode’s capacity of 170 mAh·g^−1^ in the same voltage range [22].

Zirconium-based NASICONs have demonstrated an excellent electrochemical performance at different current rates. NaZr_2_(PO_4_)_3_ delivered a specific capacity of 112 mAh·g^−1^ at a current density of 500 mA·g^−1^, between 0.01 and 2.5 V vs. Na^+^/Na [23]. The material’s high Na-ion mobility through the PO_4_-ZrO_6_ polyhedra chain enables excellent rate performance and ionic conductivity. Moreover, the substitution of Zr^4+^ cations by Magnesium and cobalt cations has shown to be a highly attractive strategy for enhancing battery performance. In the earlier studies by Feltz and Barth, the conductivity of Na_3_MZr(PO_4_)_3_ (M: Mn, Mg, Zn) NASICON-type materials synthesized using a simple solid-state method was reported, although no electrochemistry results were investigated [24]. Subsequently, in another study [25], Na_3_MnZr(PO_4_)_3_ and Na_3_NiZr(PO_4_)_3_ were synthesized through a solid-state approach, and their electrochemical properties in Na-ion batteries were tested. While Na_3_MnZr(PO_4_)_3_ was reported to be nearly inactive electrochemically, likely due to its poor kinetics or the instability of Mn^3+^ after Na extraction, Na_3_NiZr(PO_4_)_3_ showed promise, with approximately one Na able to be extracted. However, the initial charge exhibited electrolyte decomposition above the voltage window, indicating an activation process, a large polarization of approximately 0.8 V, and an average potential of 3.9 V vs. Na^+^/Na associated with Ni^3+/^Ni^2+^. In contrast, Goodenough studied the NASICON-structured Na_3_MnZr(PO_4_)_3_ as a cathode for sodium batteries, which demonstrated superior electrochemical performance compared to other manganese phosphate cathodes in the literature. Both the Mn^4+/^Mn^3+^ and Mn^3+/^Mn^2+^ redox couples were reversibly accessed in Na_3_MnZr(PO_4_)_3_, providing high discharge voltage plateaus at 4.0 and 3.5 V, respectively [26]. Recently, in our previous work, we succeeded to demonstrate the activity of Na_3_NiZr(PO_4_)_3_ prepared via the sol–gel method as an anode material for SIBs at a low voltage of 0.01–2.5 V, achieving an initial discharge capacity of 140 mAh·g^−1^ at 25 mA·g^−1^ and relatively high stability at high rates [27]. This highlights the potential of Na_3_NiZr(PO_4_)_3_ as a promising candidate for SIBs, showcasing its electrochemical performance and stability under challenging conditions.

In this study, we present novel insights into the utilization of Na_3_NiZr(PO_4_)_3_ as an anode material for sodium-ion batteries (SIBs). While our preliminary work showcased its electrochemical activity, this manuscript delves deeper into the underlying kinetics and mechanisms governing its electrochemical behavior. Through methodological advancements, we have refined our experimental and analytical approaches to offer deeper insights into the material’s electrochemical behavior, going beyond the surface-level observations of our initial study. This includes comprehensive studies on diffusion and charge transfer kinetics, providing a nuanced understanding of the material’s performance. Additionally, we have incorporated new data from extended life cycling tests and evaluations at higher rates, significantly expanding the empirical basis for our conclusions. Such rigorous testing demonstrates the material’s robustness and suitability for practical applications, a facet not explored in our preliminary communication. Moreover, employing in situ X-ray diffraction (XRD) analysis, we delve into the mechanisms of action for Na_3_NiZr(PO_4_)_3_ within SIBs, uncovering novel insights into its operational dynamics. This mechanistic exploration reveals the reasons behind the material’s high performance, addressing unexplored aspects and presenting outcomes that significantly advance our understanding.

## 2. Experimental Section

### 2.1. Material Preparation and Characterization

The precursors used in the sol–gel reaction to produce the NASICON material Na_3_NiZr(PO_4_)_3_ were NiCl_2_·6H_2_O (Sigma-Aldrich, St. Louis, MO, USA, ≥99%), ZrOCl_2_·8H_2_O (Sigma-Aldrich, ≥99%), (NH_4_)_2_HPO_4_ (Sigma-Aldrich, ≥99%), Na_2_CO_3_ (Sigma-Aldrich, ≥99%), and citric acid (as a gelling agent). The mixture was heated and agitated to 60 °C until complete evaporation and gel formation occurred. Subsequently, the resulting gel was heated in an air-filled muffle furnace at 750 °C for ten hours. To enhance conductivity, a carbon coating was applied to the NASICON material. An 85% weight percent of the powder obtained was mixed and ground with 15% weight percent of sucrose (Sigma-Aldrich, ≥99%) in pure acetone. The mixture was then subjected to calcination at 600 °C for 5 h under argon flow. X-ray diffraction (XRD) characterization was performed using a Bruker D8 advanced machine equipped with a Cu Kα radiation source. The XRD pattern was collected in the range 10–80° (2θ) in a continuous scan mode with a step size of 0.01° at room temperature. The morphology, particle size, and energy dispersion spectroscopy (EDS) mapping of the material before and after coating were observed using Zeiss Evo10 scanning electron microscopy (SEM, Munich, Germany). Raman spectrum measurements were carried out at a 532 nm wavelength incident laser light (Horiba Raman spectrometer, LabRAM HR, Vénissieux, France). The carbon content was determined using a thermogravimetric analysis method (Discovery TGA, TA instruments, New Castle, DE, USA). The thermal degradation of the carbon coating was evaluated under oxygen flow, from 25 °C to 700 °C with a heating rate of 10 °C·min^−1^.

### 2.2. Material Preparation and Characterization

A solution was prepared by combining 75 wt.% of the active substance, 20 wt.% of carbon black as a conductive agent, and 5 wt.% of Carboxy Methyl Cellulose (CMC) binder in a small quantity of deionized water. This mixture was stirred continuously overnight. Subsequently, it was evenly spread onto a substrate using a doctor blade machine and then dried in an oven for five hours at 80 °C. Circular disks with an 11 mm diameter were cut from the dried material and subjected to overnight vacuum drying at 80 °C.

For the assessment of electrochemical performance, a CR2032 coin cell configuration was employed. The synthetic electrode material was used as the anode, while sodium foil served as the counter and reference electrode. These components were assembled within a glove box filled with argon (Jacomex, Dagneux, France). A small glass fiber membrane (Whatman^®^, VWR, Briare, France), soaked with an electrolyte, was used as the separator and was placed between the two electrodes (anode and sodium foil). The electrolyte consisted of a 1 mol·L^−1^ NaPF_6_ solution dissolved in a mixture of ethylene carbonate (EC) and dimethyl carbonate (DMC) at a 50:50 volume ratio. Electrochemical measurements were conducted at room temperature utilizing a multi-channel potentiostat (MPG-2, Bio-Logic SAS, Seyssinet-Pariset, France).

For operando XRD measurements, the active material was mixed with carbon black in a weight ratio of 7:3 and assembled into an in situ cell equipped with a Beryllium (Be) window. The electrolyte used in this setup consisted of a 1 mol·L^−1^ solution of NaPF6 dissolved in a 1:1 ethylene carbonate (EC)/dimethyl carbonate (DMC) mixture. The counter electrode and separator employed were the same as those used in the electrochemical tests with coin cells.

## 3. Results

### 3.1. Synthesis and Crystal Structure of Na_3_NiZr(PO_4_)_3_

**Figure 1:**
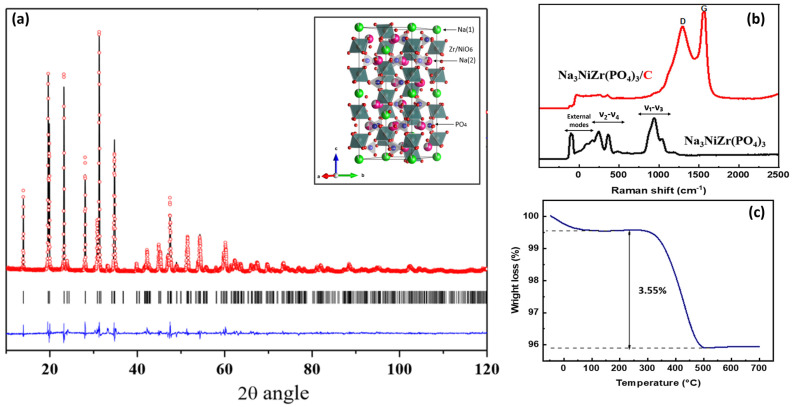
(**a**) Rietveld refinement of the as-prepared Na_3_NiZr(PO_4_)_3_ material that is indexed as the R3c symmetric space group, inset: crystal structure of the prepared material Na_3_NiZr(PO_4_)_3_; (**b**) Raman spectra of uncoated and coated sample of Na_3_NiZr(PO_4_)_3_; (**c**) TGA analysis of Na_3_NiZr(PO_4_)_3_ material.

The Rietveld refinement analysis of the X-ray diffraction (XRD) pattern confirmed the successful formation of Na_3_NiZr(PO_4_)_3_ with the rhombohedral NASICON structure within the R-3c space group. The presence of sharp peaks in the XRD pattern (Figure 1a) signifies the high crystallinity of the synthesized material. Within the R-3c space group, which is characteristic of the NASICON family, the Zr(Ni), P, and O atoms are positioned at the (12c), (18e), and (36f) Wyckoff positions, respectively. Initially, all M(1) sites were fully occupied by Na, with excess sodium (two atoms) being located at the M(2) site (18e). These adjustments resulted in a remarkably close agreement between the experimental and computed XRD patterns, as evidenced by the following reliability factors [Rp = 7.29% and Rwp = 9.3%]. The Na_3_NiZr(PO_4_)_3_ structure consists of ZrO_6_ and NiO_6_ octahedra, all of which share their corners with PO_4_ tetrahedra (inset: Figure 1a). This structural arrangement imparts exceptional stability and inherent safety to Na_3_NiZr(PO_4_)_3_, thanks to its polyanionic phosphate framework. The summary of the various structural parameters is outlined in Table S1 (supporting information). In Figure S1, within the supporting information, the X-ray diffraction (XRD) patterns of both the Na_3_NiZr(PO_4_)_3_ powders before and after the coating process are displayed. Notably, the XRD pattern of the Na_3_NiZr(PO_4_)_3_ powder is nearly identical to that of the coated powder Na_3_NiZr(PO_4_)_3_/C. This similarity strongly suggests that the coating process had no discernible impact on the structural stability of the material. Furthermore, Raman spectroscopy was employed to validate the presence of all NASICON structure-related modes, as depicted in Figure 1b. Additionally, it was used to confirm the existence of D and G bonds, which are indicative of the characteristics of the carbon coating and its application enhancing the electronic conductivity of this phosphate-based material. All vibrational modes associated with the NASICON structure were identified in the uncoated material. In the Raman spectrum of pristine Na_3_NiZr(PO_4_)_3_, the observed vibrational modes offer profound insights into the compound’s intricate molecular structure. The prominent bands resulting from the vibrations of the P-O-P and P-O bonds in the [PO_4_] tetrahedra highlight the intricate vibrational characteristics of the compound, reflecting its structural design. These spectral features, indicative of the characteristic vibrational modes associated with phosphate groups and metal-oxygen bonds, elucidate the compound’s structural integrity and electronic properties. Notably, the presence of low-frequency peaks below 200 cm^−1^ (suggestive of lattice modes) and mid-frequency range peaks between 200 and 600 cm^−1^ (representing symmetric (ν2) and asymmetric (ν4) bending modes of phosphate groups) underscores the compound’s robust crystalline framework. Furthermore, the higher frequency peaks in the 900–1200 cm^−1^ range, attributed to symmetric (ν_1_) and asymmetric (ν_3_) stretching vibrations of the P-O bonds, align seamlessly with the reported observations for analogous compounds, reaffirming the inherent stability and characteristic vibrational features of phosphate-based materials [28,29]. The coated Raman spectra of the Na_3_NiZr(PO_4_)_3_/C material displays two broad bands and strong intensities between 1350 cm^−1^ and 1580 cm^−1^, which are attributed to the vibration modes of crystalline graphite (G-band) and disordered graphite (D-band). The intensity of the G band around 1580 cm^−1^ (which corresponds to the sp2-bonded carbon atoms in a hexagonal graphitic ring) and the intensity of the D band around 1350 cm^−1^ (which corresponds to the vibrations of the sp3-bonded carbon atoms of defects and disorder) are used to describe the degree of graphitization of a carbon material in general. The obtained ID/IG ratio is around 1.17, which indicates the low graphitized carbon coating due to the low calcining temperatures. In order to quantify the amount of carbon integrated in this phosphate-based material, the thermogravimetric analysis (TGA) technique was employed as a complementary analysis method to Raman spectroscopy. As depicted in Figure 1c, it was observed that the carbon content constituted roughly 3.55% of the overall weight. This amount excludes the contribution of the amorphous carbon to the rate performance of Na_3_NiZr(PO_4_)_3_/C [26].

**Figure 2:**
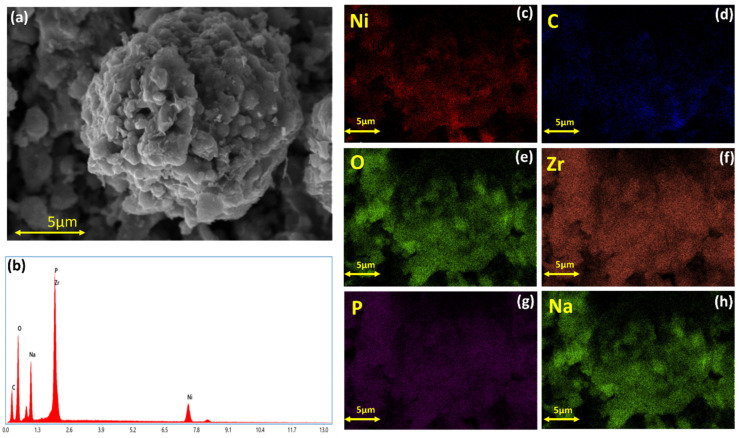
(**a**) SEM image; (**b**) EDX spectrum of areas; (**c**–**h**) element mapping images of Na_3_NiZr(PO_4_)_3_/C material.

The morphology aspect of Na_3_NiZr(PO_4_)_3_/C was examined by SEM images and EDX mapping. Figure 2a shows the image of the coated sample. It presents well-shaped and spherical microparticles with an average particle size of about 7–12 µm that are formed by the agglomeration of nanosized particles. As for the uncoated material, the particles have an irregular form in addition to the presence of some agglomerations, as shown in Figure S2. To obtain chemical information detailing the elemental distribution of the Na_3_NiZr(PO_4_)_3_/C, we carried out energy dispersive X-ray spectroscopy (EDX)-based mapping analysis. The EDX analysis suggests that elements Na, Ni, P, and Zr were uniformly distributed throughout the area under study, as seen in Figure 2c–h.

### 3.2. Electrochemical Performances

**Figure 3:**
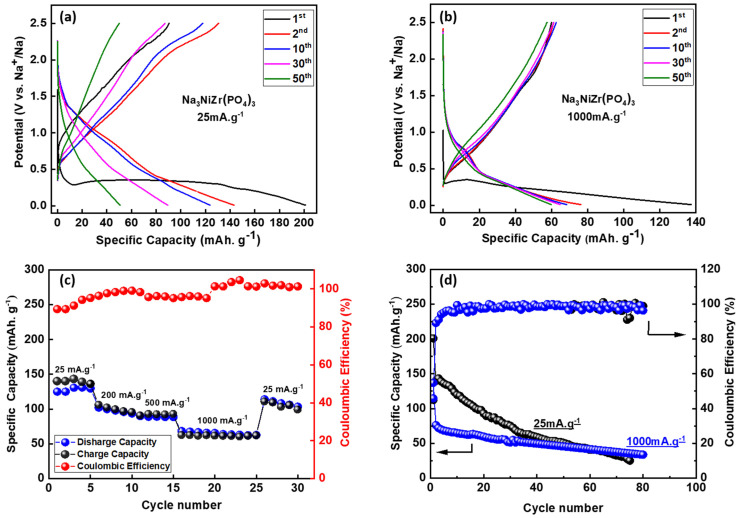
(**a**) discharge/charge profiles of Na_3_NiZr(PO_4_)_3_/C material at 25 mA·g^−1^; (**b**) discharge/charge profiles of Na_3_NiZr(PO_4_)_3_/C material at 1000 mA·g^−1^; (**c**) Rate capability of Na_3_NiZr(PO_4_)_3_/C tested at different current densities 25, 200, 500 and 1000 mA·g^−1^; (**d**) capacity retention of Na_3_NiZr(PO_4_)_3_/C cycled at 25 and 1000 mA·g^−1^.

**Figure 4:**
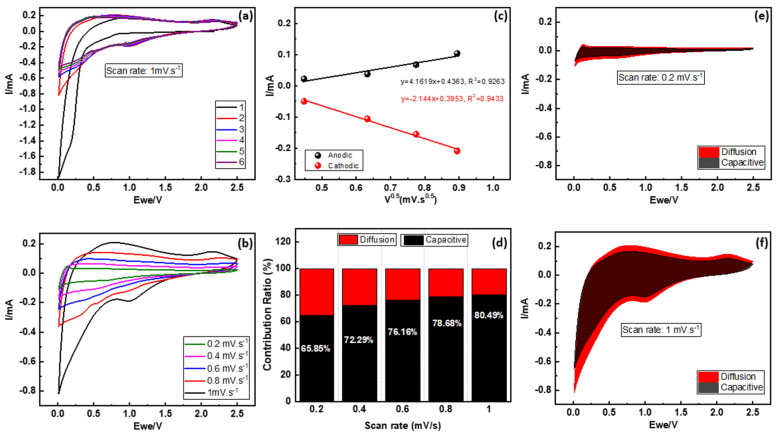
(**a**) Cyclic voltammograms of Na_3_NiZr(PO_4_)_3_/C at a scan rate of 1 mV·s^−1^; (**b**) Cyclic voltammograms curves at different scanning rates; (**c**) The relationship between the peak current (Ip) and the square root of the scan rate (ν 0.5); (**d**) contribution ratio of the controlled capacitive and diffusion currents at various scan rates; (**e**) CV curve with capacitance contribution at scan rate 0.2 mV·s^−1^; (**f**) CV curve with capacitance contribution at scan rate 1 mV·s^−1^.

In Figure 3a, the electrochemical performance of Na_3_NiZr(PO_4_)_3_/C is showcased, revealing exceptional outcomes within the voltage window of 0.01 to 2.5 V versus Na^+^/Na at a current density of 25 mA·g^−1^. Notably, the first cycle exhibits a remarkably high discharge capacity, delivering 200 mAh·g^−1^ and presenting a distinct plateau shape comprising a characteristic absence in subsequent cycles. The stability of the capacity becomes evident from the second cycle onward, maintaining a specific discharge capacity of 141 mAh·g^−1^ until the tenth cycle. Intriguingly, a sudden decline to 50.77 mAh·g^−1^ occurs after 50 cycles. Unlike its behavior at lower rates, Na_3_NiZr(PO_4_)_3_/C demonstrates enhanced stability at high rates, as elucidated by the galvanostatic charge/discharge profiles recorded at 1000 mA·g^−1^ (Figure 4b). Remarkably, the material conserves its initial behavior in the first cycle at high rates, presenting a plateau shape as well and a notable discharge capacity of 140 mAh·g^−1^. Then, the capacity stabilizes at nearly 62 mAh·g^−1^. This observed phenomenon aligns with prior findings in analogous materials, suggesting structural rearrangements during discharge to low voltages (≤0.5 V) [20,28]. The enhanced capacity in this cycle in both rates is attributed to the formation of the solid electrolyte interface (SEI) and the significant contribution of carbon black a crucial factor for improving of the electrochemical performance of such materials [30,31]. The rate capability test, as illustrated in Figure 4c, further substantiates the material’s prowess, exhibiting discharge and charge profiles at varying rate capabilities of 25, 200, 500, and 1000 mA·g^−1^. Impressively, the material delivers discharge capacities of 143, 106, 102, and 76 mAh·g^−1^, respectively (initial charge and discharge curves depicted in Figure S3). Importantly, the material is underscored by its consistent capacity retention with a specific discharge capacity of 101 mAh·g^−1^ even after reverting to 25 mA·g^−1^. This exceptional rate capability is corroborated by a long-term cycling test presented in Figure 4d, where the material demonstrates stability from the second cycle to the eightieth cycle, maintaining a specific charge capacity of 25 and 36 mAh·g^−1^ after eighty cycles at 25 and 1000 mA·g^−1^, respectively. The low initial coulombic efficiency of 45% is attributed to factors such as electrolyte decomposition, side reactions at lower current densities, and electrochemical polarization [32] providing valuable insights into the material’s electrochemical behavior. High coulombic efficiency of 96% and 98% for both rates of 25 and 1000 mA·g^−1^, respectively, after 80 cycles underscores the robustness of the material, showcasing the high reversibility of cycles in both cases.

NASICON-type structure, known for its facilitative three-dimensional network for Na^+^ ion diffusion, plays a crucial role in its functionality. However, our analysis concurs that intrinsic characteristic, such as the crystal structure and ion transport pathways, significantly contribute to the observed low capacity and fade in performance over time, especially under low-rate conditions. The cyclic stress induced by the continuous intercalation and deintercalation of Na^+^ ions during charge–discharge cycles can compromise the structural stability of Na_3_NiZr(PO_4_)_3_, potentially leading to degradation or phase changes that deteriorate its performance. Additionally, the inhomogeneous distribution of Na^+^ ions exacerbate performance fade, as it results in uneven stress and strain within the crystal structure, further promoting structural degradation [11]. These insights underscore the need for targeted strategies to mitigate these structural challenges, thereby enhancing the durability and efficiency of Na_3_NiZr(PO_4_)_3_ for energy storage applications.

To interpret these conducts and the high-rate performance of this material, cyclic voltammetry measurements was performed to analyze the kinetics as presented in Figure 4. Upon initial inspection at the scanning rate of 1 mV·s^−1^ in the voltage window 0.01–2.5 V, the first scan is markedly different from the subsequent cycles, indicating an activation process or the formation of a solid electrolyte interphase (SEI) layer, which explains the first low coulombic efficiency in the GCPL test, as shown before in Figure 3d. For the subsequent cycles, the cathodic portion is manifested at 0.8–1.2 V, while the anodic portion appears at 2.2–2.4 V. The absence of any notable distinction from the second cycle to the sixth cycle, as depicted in Figure 4a, underscores the stability of the Na_3_NiZr(PO_4_)_3_/C material and supports the concept of a reversible redox process for sodium-ion reactions. The third pair of redox peaks can be found around 0 V, which is unusual. This pair is attributed to Na^+^ insertion/extraction from the interlayer of the graphite micro-crystallites, according to earlier study findings on Na+ storage properties in carbonaceous materials [14,15]. Notably, nearly all of the CV profiles overlap on successive cycles, indicating excellent electrochemical reversibility. In Figure 4b, CV profiles were obtained at various sweep speeds ranging from 0.2 to 1 mV·s^−1^ to gain a better understanding of the electrochemical kinetics of Na^+^ (de)insertion into the Na_3_NiZr(PO_4_)_3_/C. The Na^+^ diffusion coefficient D can be determined using the Randles–Sevcik Equation (1) by fitting the linear connection between peak currents and the square root of the scan rate for anodic and cathodic peaks (Figure 4c).

(1) $$I_{p}=2.69\;\times \;10^{5}n^{3/2}{\mathrm{AD}}^{1/2}{\mathrm{Cv}}^{1/2}$$

where I_p_ is the cathodic or anodic peak current, n is the number of electrons per molecule during intercalation (n = 2), A is the effective contact area between the electrode and the electrolyte, C is the Na^+^ concentration in the electrode, D is the Na^+^ diffusion coefficient, and v is the scan rate. It should be noted that A is chosen as the geometric area of the electrode in this study, 0.95 cm^2^, for simplicity, as is customary in the literature. As a result, for A and C, D may be computed as 7.30716 × 10^−7^ cm^2^·s^−1^ and 2.75348 × 10^−6^ cm^2^·s^−1^, respectively. These computed values are a little higher from those of NaTi_2_(PO_4_)_3_/C [33], Na_3_V_2_(PO_4_)_3_/C [34] and other compounds. To elucidate the charge storage mechanism within the experimental framework and to quantify the respective contributions of capacitive and diffusion-limited processes to the aggregate capacity, the investigative approach delineated by Dunn and colleagues was adopted. This analytical method predicates the assessment of the current at a designated voltage, with the ensuing contributions being apportioned to capacitive (denoted as k_1_) and diffusion-controlled (denoted as k_2_) processes. This methodology facilitates a comprehensive dissection of the charge storage dynamics, thereby enabling a more nuanced understanding of the interplay between capacitive and diffusion mechanisms in influencing the total capacity of the system under investigation.

i = k_1_v + k_2_v^1/2^ (2)

At a scanning rate of 0.2 mV·s^−1^, the capacitive component of the charge storage is identified to be 66%. When the scanning rate is adjusted to 0.4, 0.6, 0.8, and 1 mV·s^−1^, the capacitive contributions are, respectively, determined to be 72%, 76%, 78%, and 81%, as demonstrated in Figure 4d. For instance, the capacitive component is measured to constitute 66%and 81% at a scanning rate of 0.2 and 1 mV·s^−1^, respectively, as depicted in Figure 4e,f. These results, indicating a predominant capacitive contribution at various scanning rates, suggest rapid kinetic reactions, which contribute to the enhanced rate performance observed in this Na_3_NiZr(PO_4_)_3_/C anode material. The capacitive performance of Na_3_NiZr(PO_4_)_3_ battery materials can be attributed to a combination of surface and bulk properties that facilitate rapid charge storage mechanisms. Primarily, the surface adsorption and desorption of sodium ions on the material’s surface contribute to a capacitive behavior, enabling fast charge and discharge processes. Additionally, the structure supports surface redox reactions involving transition metals, such as nickel, which further enhance capacitive effects. The microstructure of Na_3_NiZr(PO_4_)_3_ provides numerous active sites for ion exchange, amplifying these capacitive characteristics. At higher charge–discharge rates, the limited diffusion in the bulk material emphasizes the significance of these surface-dominated processes, leading to the observed capacitive behavior [20,35]. This combination of factors, supported by cyclic voltammetry analysis, underlines the role of both surface reactions and microstructural features in the capacitive performance of NZP battery materials. The remarkable stability and outstanding performance achieved at such a high rate can be ascribed to several key factors. Firstly, the Na_3_NiZr(PO_4_)_3_, with its NASICON structure, exhibits an exceptionally stable and open framework, promoting swift Na^+^ diffusion and enhancing cycling stability. Additionally, the absence of voltage plateaus and the consistently high average voltage values effectively address the issue of sodium plating, which is a common concern in advanced anode materials such as hard carbon. The above-mentioned variables work together to provide Na_3_NiZr(PO_4_)_3_ with exceptional structural stability and enhanced charge transfer. Consequently, this leads to the attainment of exceptional high-rate performance and enduring cycling stability. These properties and performance make this material a good candidate for both stationary and fast charging applications [22]. However, to improve the specific capacity and energy density, more research is needed in the future, including doping, nanocrystallization, and the hunt for NASICON structural analogs [11,36,37].

### 3.3. In Situ XRD of Na_3_NiZr(PO_4_)_3_/C

**Figure 5:**
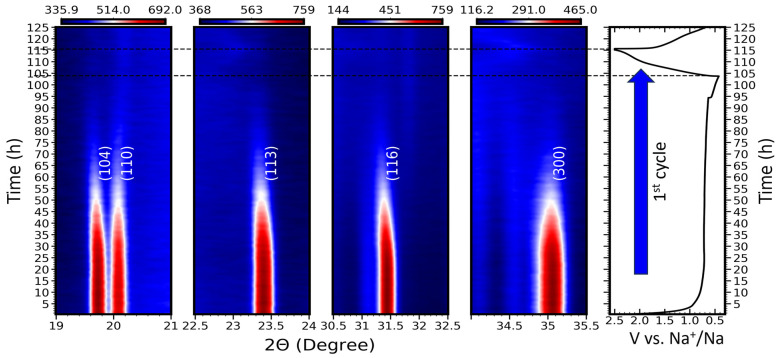
In situ XRD patterns collected during charging and discharging of Na_3_NiZr(PO_4_)_3_/C half-cell at the voltage range of 0.01 and 2.5 V vs. Na^+^/Na.

In situ XRD was used to obtain valuable information about the structural modifications occurring along the insertion process of sodium, where X-ray diffraction patterns within a two theta angle that ranged from 10° to 40° were recorded while the material was cycled in the voltage window between 0.01 and 2.5 V versus Na^+^/Na, as seen in Figure 5. All the diffraction patterns observed at the open-circuit voltage state can be attributed to the rhombohedral phase of Na_3_NiZr(PO_4_)_3_. The sodium insertion inside the structure causes a gradual weakening of all the peaks of the rhombohedral phase without the creation of new peaks, which matches the amorphization of the material. The rhombohedral phase disappears completely in the middle of the discharge, and no new peaks are observed at the end of the first discharge [33,34]. In the first charge and second discharge, the current density was increased in order to have a faster cycle. The material appears to stay in an amorphous state with the variation in the number of sodium ions. A slight non-assigned peak shift occurs around 22.75° and 34.5° with the desodiation and sodiation of the material. When the electrode is discharged to a voltage lower than 0.1 V, the Na_3_NiZr(PO_4_)_3_ material’s NASICON-like crystalline characteristics diminish. Subsequently, even after recharging the Na_3_NiZr(PO_4_)_3_ electrode to 2.5 V, the NASICON structure remains unrecovered. This alteration in the arrangement of the structure can be identified as the primary factor behind the irreversible nature of the initial discharge pattern depicted in Figure 5. Additionally, the inset of multi rate cyclic voltammetry test shown before displays the initial six cyclic voltammograms for the Na_3_NiZr(PO_4_)_3_ electrode, spanning from 0.01 to 2.5 V, utilizing a deliberately scan rate of 0.01 mV·s^−1^. These obtained curves indeed validate the irreversibility of the first discharge profile, as also observed in the galvanostatic tests conducted at a low current density of 25 mA·g^−1^. The notable high discharge capacity witnessed during the initial cycle could suggest the participation of an electrochemical mechanism linked to Na^+^ intercalation reactions, as previously reported. This is akin to a discharge voltage of 0.01 V, which corresponds to the findings reported for Mg_0.5_Ti_2_(PO_4_)_3_ [38]. This correlation implies a greater involvement of sodium ions in the reaction, consequently leading to an augmented discharge capacity. These outcomes are in alignment with the earlier materials documented in the literature, which are listed in Table S2 [20,28,29,39,40].

**Figure 6:**
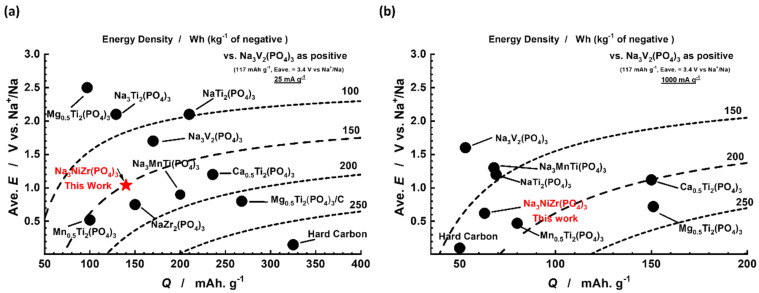
Comparative map of phosphate-based anode materials for SIBs at (**a**) 25 mA·g^−1^ and (**b**) 1000 mA·g^−1^.

A representative comparison of the energy density, discharge capacity, and average potential vs. Na^+^/Na was carried out for various NASICON anode materials previously reported in the literature. The majority of these NASICON compounds shown in Figure 6a have a different average potential and a distant charge capacity but reveal each one of their special characteristics. Na_3_Ti_2_(PO_4_)_3_ [41] and NaTi_2_(PO_4_)_3_ [33] have a similar average voltage of 2.1 V vs. Na^+^/Na, but also have a large difference in energy density and charge capacity as well. In the meantime, among titanium-based anode materials, Mg_0.5_Ti_2_(PO_4_)_3_ [38] presents the highest average potential that an anode can present of 2.5 V vs. Na^+^/Na. However, a carbon-coated Mg_0.5_Ti_2_(PO4)_3_/C [20], reported by Zhao et al., demonstrates different electrochemical properties with an lower average potential of 0.8 V vs. Na^+^/Na, and a higher specific capacity of 268.6 mAh·g^−1^. Additionally, Ca_0.5_Ti_2_(PO_4_)_3_ [21], Mn_0.5_Ti_2_(PO_4_)_3_ [42], and NaZr_2_(PO_4_)_3_ [23] exhibit a lower average potential compared to previous ones with a values of 1.2, 0.52, and 0.75 V vs. Na^+^/Na, respectively. Among these materials, hard carbon stands out with the lowest average potential and an impressive specific charge capacity of 325 mAh·g^−1^ at 0.1C as well as an excellent energy density [43]. However, it presents relatively poor rate performance that is caused by sodium-ion diffusion that takes place along the channels and cavities with irregular geometries [43,44]. As seen in Figure 6b, hard carbon shows a low discharge capacity and lower energy density compared to NASICON materials presented in the same figure. Moreover, it shows low average voltage, which raises the problem of the sodium plating causing a serious safety issue. On the other hand, NASICON materials exhibit high performance at high rates, showing acceptable capacity and good average voltage vs. Na^+^. Although hard carbon exhibits remarkable performance as an anode material, its low average potential raises safety concerns due to the issue of sodium plating in SIBs, and its low-rate performance makes it less favorable for applications demanding high-power output and rapid energy delivery. In contrast, Na_3_NiZr(PO_4_)_3_/C exhibits a higher average potential of 0.68 V vs. Na^+^/Na compared to hard carbon and moderate discharge capacity and energy density as well. These characteristics make Na_3_NiZr(PO_4_)_3_/C a good choice when it comes to safety concerns and fast energy applications.

## 4. Conclusions

This study presents Na_3_NiZr(PO_4_)_3_/C as a potential candidate for use in Na-ion batteries. The material was synthesized using a sol–gel technique and was then thermally treated in an air environment at 750 °C. The results showed that Na_3_NiZr(PO_4_)_3_ has a rhombohedral NASICON structure and belongs to the space group R-3c. The inclusion of carbon coating was further validated during synthesis using Raman spectroscopy, which found typical bands associated with this incorporation, and was quantified via thermogravimetric analysis. Using scanning electron microscopy (SEM), it was discovered that the particle sizes of Na_3_NiZr(PO_4_)_3_/C were on the nanometric scale. The electrochemical performance was evaluated over a voltage range of 0.01 to 2.5 V. The initial discharge capacity of Na_3_NiZr(PO_4_)_3_/C was measured at 200 mAh·g^−1^ at a current density of 25 mA·g^−1^. However, the capacity decreased steadily during repeated charge–discharge cycles. Despite this initial capacity loss, the anode material performed impressively at high current rates and exhibited long-term cycling stability and a high coulombic efficiency of over 98%. The enhanced electrochemical performance of this material is a result of its superior Na^+^ diffusion coefficient when compared with its analogues and the contribution of the capacitive behavior. The findings of this study were supported by operando diffraction, which provided vital insights into the structural changes that occurred throughout the sodium insertion process. As a result, Na_3_NiZr(PO_4_)_3_/C appears as an intriguing candidate for the construction of fast and safe rechargeable sodium batteries.

## Data Availability

Data are contained within the article and Supplementary Materials.

## Supplementary Materials

The following supporting information can be downloaded at: https://www.mdpi.com/article/10.3390/nano14141204/s1, Table S1. Atomic positions of Na_3_NiZr(PO_4_)_3_ from Rietveld refinement of X-ray diffraction pattern (Rp = 7.29%; Rwp = 8.3% and Rexp = 4.02%); Figure S1. XRD pattern of Na_3_NiZr(PO_4_)_3_ uncoated material (a) and coated material (b); Figure S2. SEM images of Na_3_NiZr(PO_4_)_3_ uncoated material; Figure S3. Initial charge and discharge curves tested at different current densities; Table S2. List of NASICON anode materials used for comparison.
