# Supplementary material for: Rate-Dependent Stability and Electrochemical Behavior of Na3NiZr(PO4)3 in Sodium-Ion Batteries
Source: Nanomaterials (Basel). 2024 Jul 16;14(14):1204. doi: 10.3390/nano14141204 (PMC11279984; doi:10.3390/nano14141204)
Supplement: Supplementary file 1 [file nanomaterials-14-01204-s001.zip › nanomaterials-2878592-supplementary.pdf]

Rate-Dependent Stability and Electrochemical Behavior of $Na_3NiZr(PO_4)_3$ in Sodium-Ion Batteries

Marwa Tayoury, Abdelwahed Chari, Mohamed Aqil, Adil Sghiouri Idrissi, Ayoub El Bendali, Jones Alami,
Youssef Tamraoui and Mouad Dahbi *

Materials Science, Energy, and Nano-engineering Department, Mohammed VI Polytechnic University,
Ben Guerir 43150, Morocco
* Correspondence: mouad.dahbi@um6p.ma

**Atom**	**Wyckoff site**	*x*	*y*	*z*	**B**	**Occ.**
**Zr/Ni**	12c	0.00000	0	0.14737	-0.044	1.0000
**Na(1)**	6b	0.63349	0	0.25000	3.942	2.0000
**Na(2)**	18e	0.00000	0	0.00000	7.178	1.0000
**P**	18e	0.28828	0	0.25000	1.158	3.0000
**O(1)**	36f	0.18561	-0.02363	0.19233	2.350	6.0000
**O(2)**	36f	0.19276	0.16776	0.08817	2.350	6.0000

Table S1. Atomic positions of $Na_3NiZr(PO_4)_3$ from Rietveld refinement of X-ray diffraction pattern (Rp = 7.29 %; Rwp = 8.3% and Rexp = 4.02%)

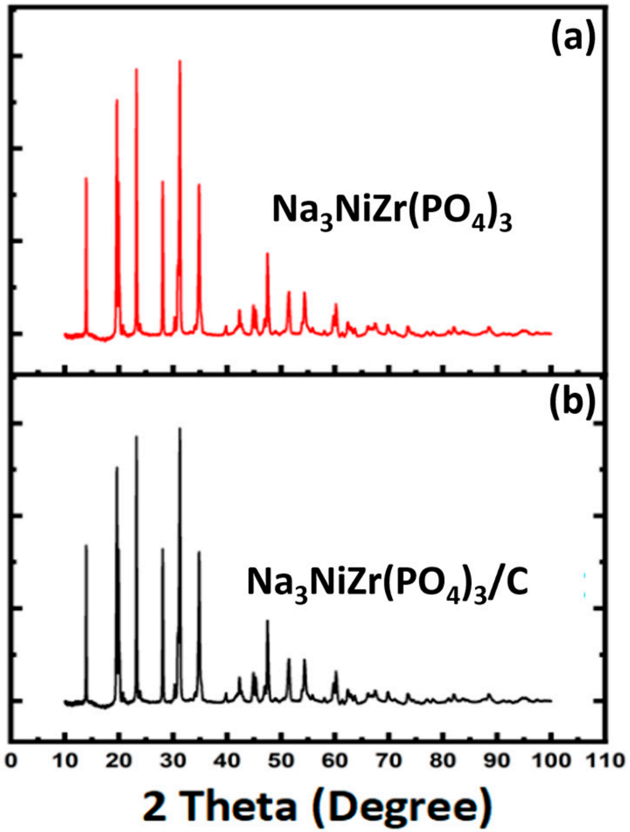

Figure S1. XRD pattern of $Na_3NiZr(PO_4)_3$ uncoated material (a) and coated material (b)

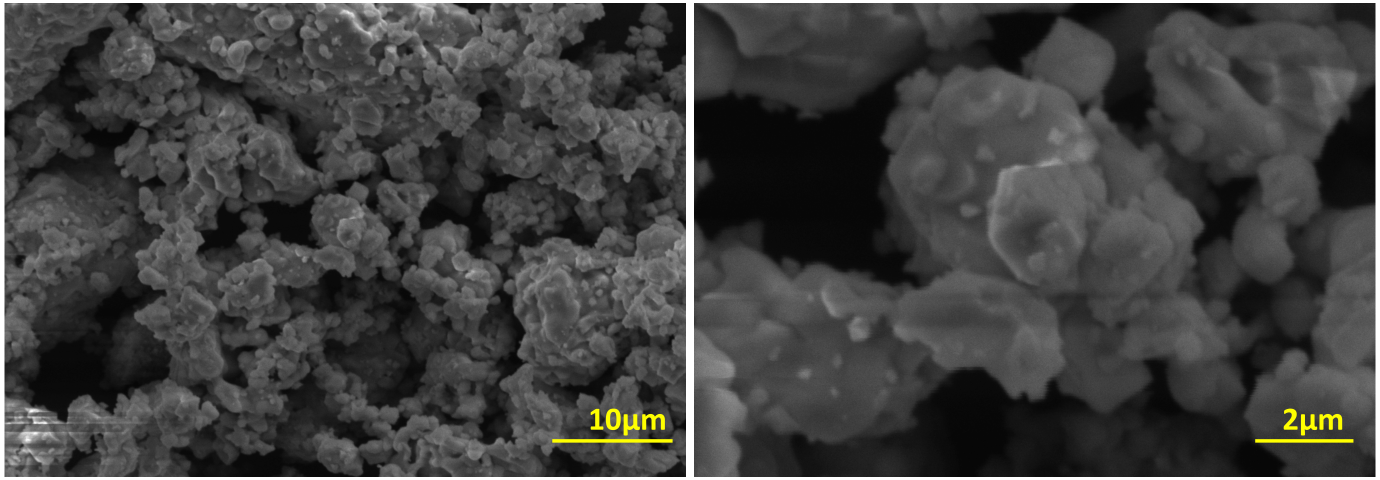

Figure S2. SEM images of $Na_3NiZr(PO_4)_3$ uncoated material

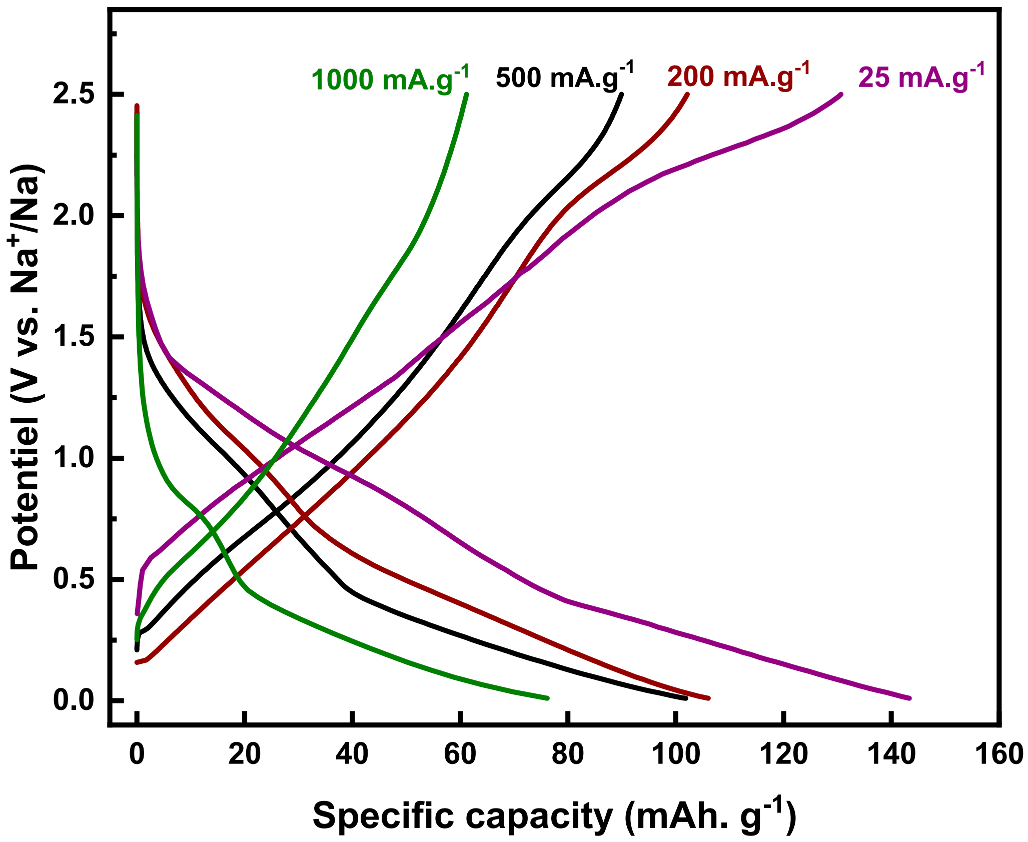

Figure S3. Initial charge and discharge curves tested at different current densities.

Material	Average voltage / V	Specific capacity / mAh.g^{-1}	Reference
$NaTi_2(PO_4)_3$	2.1	210	[19]
$Na_3Ti_2(PO_4)_3$	2.1	129	[7]
$Na_3V_2(PO_4)_3$	1.6	170	[18]
$NaZr_2(PO_4)_3$	0.75	150	[10]
$Mn_{0·5}Ti_2(PO_4)_3$	0.52	100	[23]
$Ca_{0.5}Ti_2(PO_4)_3$	1.2	236	[22]
Hard carbon	0.15	325	[8]
$Mg_{0.5}Ti_2(PO_4)_3$	2.5	97	[20]
$Mg_{0.5}Ti_2(PO_4)_3/C$	0.8	268	[21]
$Na_3NiZr(PO_4)_3$	1.04	143	Our work

Table S2. List of NASICON anodes materials used for comparison.
